# An Overview of the Impact of Pharmaceuticals on Aquatic Microbial Communities

**DOI:** 10.3390/antibiotics11121700

**Published:** 2022-11-25

**Authors:** Isabel Pinto, Manuel Simões, Inês B. Gomes

**Affiliations:** 1 LEPABE—Laboratory for Process Engineering, Environment, Biotechnology and Energy, Faculty of Engineering, University of Porto, Rua Dr. Roberto Frias, 4200-465 Porto, Portugal; 2 ALiCE—Associate Laboratory in Chemical Engineering, Faculty of Engineering, University of Porto, Rua Dr. Roberto Frias, 4200-465 Porto, Portugal

**Keywords:** antimicrobial resistance, biofilms, ecological interactions, emerging contaminants, microbial diversity, one health, virulence

## Abstract

Pharmaceuticals are present as pollutants in several ecosystems worldwide. Despite the reduced concentrations at which they are detected, their negative impact on natural biota constitutes a global concern. The consequences of pharmaceuticals’ presence in water sources and food have been evaluated with a higher detail for human health. However, although most of the pharmaceuticals detected in the environment had not been designed to act against microorganisms, it is of utmost importance to understand their impact on the environmental native microbiota. Microbial communities can suffer serious consequences from the presence of pharmaceuticals as pollutants in the environment, which may directly impact public health and ecosystem equilibrium. Among this class of pollutants, the ones that have been studied in more detail are antibiotics. This work aims to provide an overview of the impacts of different pharmaceuticals on environmental biofilms, more specifically in biofilms from aquatic ecosystems and engineered water systems. The alterations caused in the biofilm function and characteristics, as well as bacteria antimicrobial tolerance and consequently the associated risks for public health, are also reviewed. Despite the information already available on this topic, the need for additional data urges the assessment of emerging pollutants on microbial communities and the potential public health impacts.

## 1. Introduction

As a consequence of population growth, urbanization and climate change, constant pressure is put on atmospheric, aquatic and terrestrial environments. Furthermore, there is a rising demand to satisfy societal and economic needs, which encouraged the launch of novel chemical substances into the market [1]. Due to higher diversity, design, production and consumption rates, their release and dispersal into the environment have skyrocketed in just over the last half-century [2]. These substances have been reported in groundwater, surface water, seawater, drinking water (DW), bottled water, wastewater (WW), precipitation, swimming pools, irrigation water, food sources, suspended solids, sediments and soil [3,4,5,6,7,8,9,10], with a diverse range of substances that can be sorted into varied categories, including pharmaceuticals, cosmetics and personal care products, pesticides, herbicides, surfactants, domestic and industrial chemicals, antibiotic-resistant genes and bacteria (ARG and ARB, respectively), endocrine disruptors, psychotropic substances, microplastics, among others [11,12,13,14,15,16,17,18,19]. Emerging contaminants (ECs) are biological agents, organic or inorganic [13,20,21] compounds of anthropogenic or natural origin [11], mostly found at trace concentrations (ng/L and µg/L) in the environment [11,20,22]. It has been estimated that general pollution can be the cause of more than 9 million deaths each year globally, and more than 90% of these deaths occur in low- and middle-income countries [23]. Moreover, in 2019 it was estimated that around 1.4 million deaths were related to water pollution; unsafe water sources were the main responsible for these deaths, representing circa 1.23 million deaths [23].

**Figure 1:**
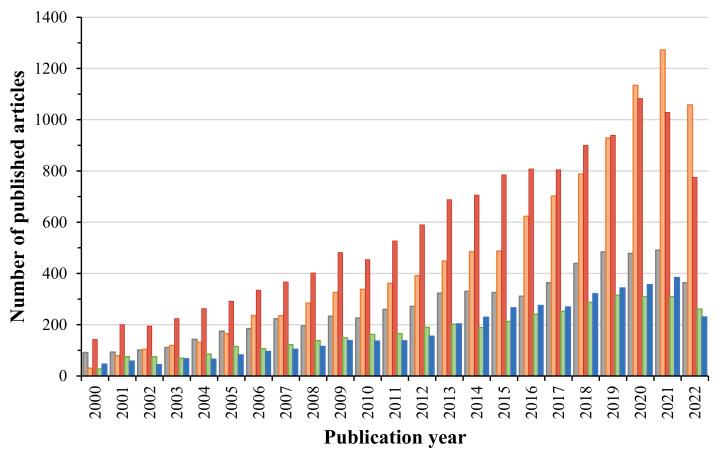
Publications reporting the occurrence of ECs in different water systems from 2000–Present (■—groundwater; ■—drinking water or tap water; ■—wastewater; ■—surface water; ■—seawater).

Along with reports of the occurrence of contaminants in the environment, the concern about unprecedented ecological repercussions arises. Recently, a global and emerging concern of the scientific community has been reflected in the possible effect of continuous exposure to residual concentrations of ECs on multiple organisms and microenvironments, leading to the study of ECs occurrence, fate, transport, effects and more. Figure 1 evidences the considerable rise in research results on the occurrence of pharmaceuticals in five different aquatic environments (groundwater, surface water, seawater, wastewater and drinking water) since the beginning of the 21st century. The data presented in Figure 1 was obtained through an advanced search in the PubMed database, by selecting titles and abstracts as search fields and by using key search words, such as Pharmaceuticals + (occurrence OR presence) + groundwater/wastewater/drinking water/surface waters (River OR Streams OR Wetlands)/seawater. The increasing number of publications reporting the presence of pharmaceutical ECs in aquatic environments, do not only demonstrate the increasing load of these contaminants in water but is also representative of the relevance of this topic and the global concern related to it. The development of a new and more precise methodology to adequately identify and quantify ECs also contributed to an increase in the number of scientific publications reporting the presence of pharmaceuticals in water over the years. Definitively, most of the published studies have focused on the presence of pharmaceutical residues in wastewater and surface water. However, in the first years of the 21st century (2000–2007), the number of published studies reporting pharmaceuticals in DW was higher or similar to those focused on the presence of pharmaceuticals in WW. However, from 2007 it is evident a significant increase in the number of articles reporting pharmaceuticals in WW each year until 2022, which may be related to the constant search for technologies able to remove ECs from WW. With suspected or recognized harmful health and environmental impact [24], these substances could pose a threat to overall human, animal and environmental health. The environmental movement of the modern era could be attributed to Rachel Carson’s book “Silent Spring,” published in 1962 [25]. Although it sparked controversy and considerable opposition at that time, Carson exposed the problematic effects of the indiscriminate use of agricultural chemicals, pesticides, and other modern chemicals, in the following years of World War II [26,27]. Her work pioneered today’s ecotoxicological studies [28].

Even though pharmaceuticals are one of the most extensively studied classes of ECs [5], information regarding the impact of antibiotics and non-antibiotic ECs on environmental microbiomes is still limited. The present work aims to provide an overview of the effects of ECs on environmental microbial communities, focusing specifically on pharmaceutical ECs’ impact on aquatic biofilms and engineered water systems and the challenges associated with these studies. This review also brings awareness to the role of antibiotics and non-antibiotics on acquired antimicrobial tolerance and dissemination of ARBs and ARGs.

## 2. Pharmaceuticals Occurrence and Exposure Pathways

**Figure 2:**
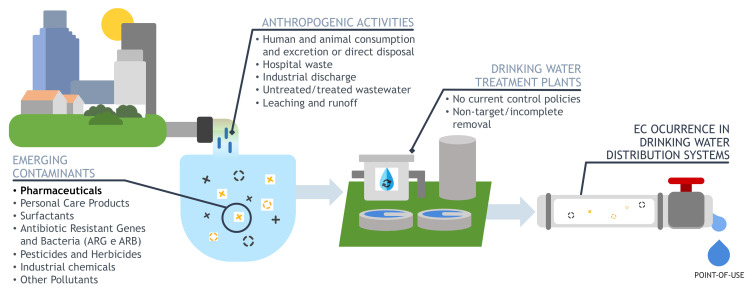
Overview of ECs sources and transport to point-of-use: Anthropogenic activities (coming from human and animal consumption and excretion; hospital waste; industrial discharge; untreated or inefficiently treated wastewater; direct disposal; landfill leaching; agricultural activities; runoff [43,44]) are the main sources of emission of these pollutants, that are not currently monitored and are usually detected at trace level concentrations in the environment. Contaminated water resources are the source of EC presence in DW, as the equipment used in WTPs does not remove these contaminants and results in treated water being redirected to DWDS. Finally, the consumer is exposed to ECs.

Pharmaceutical compounds are synthetic or natural chemicals found in prescription or over-the-counter medications destined for human or animal use [17,29]. There are currently over 20,000 approved prescription drugs on the market [30], and their extensive and abusive use substantially contributed to their dispersal in the environment, being detected in drinking water treatment plants (DWTPs) and wastewater treatment plants (WWTPs), seawater, sediments, surface waters and groundwater [29,31,32,33]. Studies have proven that no water environment is free of pollutants, regardless of its source [34] or sampling time [35]. Succinctly, the main point sources of emission are untreated and treated wastewater, as equipment and processes used in DWTPs and WWTPs do not fully, specifically (target) and effectively eliminate most of the ECs present in their influents [22,29,36]. Furthermore, hospital waste, livestock activities, aquaculture, human and animal excretion, and improper disposal of expired medical prescriptions [37,38,39] are also point sources of emission. Other non-point sources are runoff from wastewater sludge discharge and stormwater runoff [39]. As pharmacological drugs contain complex structures and varied biological and physicochemical properties [29,40], their stability and resistance to biodegradation or water solubility influence the effectiveness of removal in water treatment plants (WTPs) [3,22,37,41]. Since groundwater and surface water are the main resources of water used for the production of water for consumption, they end up becoming sources of ECs in DW [3,29,42]. Figure 2 illustrates the sequence of events stated above, demonstrating how ECs reach water bodies and make their way into DW.

Additionally, ECs concentrations in different water resources may vary seasonally and culturally, depending on the region [11,12], as well as in WWTP influents and effluents, due to factors such as production, sale and consumption rate of these compounds (both by humans and animals); on metabolism and rate of excretion [11]; and their persistence (governed by biodegradation, photolysis, and other abiotic transformations, such as hydrolysis [45]). All these factors contribute to significant variations in concentration among compounds. Special attention should also be paid to other factors that contribute to the increase of their concentration in the environment, such as bioaccumulation and biomagnification [5]. Also, ECs do not need to be persistent in the environment to cause accumulation and increase in the environment since the rates of transformation and removal of pharmaceuticals are generally offset by their constant introduction into aquatic systems [46]. Furthermore, other environmental events also contribute to this variation, such as rainfall (greater flow of water into water resources and dilution of ECs) and low temperatures (reduced biodegradation activity) [3]. In the case of DWDS, another factor that can influence and reduce concentrations is the reaction between ECs and disinfecting agents (chlorine, chloramine, and chlorine dioxide, among others) [47]. Despite the widespread of ECs in water sources worldwide, there are no specific data related to the impact of pharmaceutical pollution on the number of global deaths, however, this kind of pollution is highly related to reproductive problems [23].

## 3. Environmental Impact of Pharmaceutical Contaminants

Ecotoxicology holistically focuses on the impact of anthropogenic activities accountable for the release of pollutant molecules into the environment and its harmful impact on organisms as individuals, populations or the biosphere as a whole [48]. Pharmaceuticals are known to be chemically stable active substances intended to trigger biological responses [29,40] in target organisms [28,40]. Yet, this stability impairs its degradation in the environment, which also causes repercussions on its activity in non-target organisms, further exacerbating the concern about water safety. Prolonged exposure to environmentally relevant concentrations (i.e., trace concentrations) of pseudo-persistent, persistent and accumulative pharmaceuticals could lead to unpredictable and undesirable outcomes [3,49] in both target and non-target organisms [50], affecting the macro or microbiome and fauna. This subject is still underexplored, even though pharmaceutical contaminants are one of the most explored subjects of all classes of contaminants in the ecotoxicology field [5]. Moreover, many pharmaceuticals undergo a series of transformations resulting in the formation of metabolites or by-products [51], some biologically active and more toxic than the parent molecule [52], therefore affecting non-target aquatic organisms [53]. Many studies describe the effects of exposure to diverse ECs on fluvial, marine, wetlands, soil, gut, and WWTPs microbiome, as well as on organisms belonging to higher levels in the biological hierarchy, whose behavioral changes provide an insight into the direct or indirect impact of residual ECs [54].

Though often neglected, pharmaceuticals at low concentrations are environmental stressors that mediate physical, behavioral and cognitive changes in aquatic organisms leading to negative repercussions in evolutionary and ecological processes [55,56]. Cerveny et al. [57] have shown that some wild fish of European watercourses are physiologically affected by exposure to pharmaceuticals from different therapeutic classes. In that study, Cerveny et al. [57] screened for the presence of 94 pharmaceuticals in fish blood plasma in water courses from the Czech Republic, Germany and the United Kingdom (UK). They found 23 different pharmaceuticals in fish blood plasma. Cerveny et al. [57] also reported that in some cases, the concentration of risperidone and flupentixol detected in fish blood plasma was higher than those reported in human blood plasma. Also, Meador et al. [58] assessed the effects of 32 days of exposure to trace concentrations of a mixture of 16 ECs in feral juvenile Chinook salmon (*Oncorhynchus tshawytscha*) on estuaries receiving WWTP effluents. This work suggested that this exposure resulted in adverse physiological effects on the fish’s metabolic status (blood chemistry values indicating metabolic stress and signals of induced starvation). Furthermore, pharmaceutical bioaccumulation in aquatic fauna has also been widely reported [59,60].

Moreover, plants have also been affected by the presence of pharmaceuticals in ecosystems. In some cases, these consequences result from alterations in soil microbiota, which will affect plant development. For example, Kovacs et al. [61] proved that the presence of common non-steroidal anti-inflammatory drugs (NSAIDs) could alter the ecosystem services provided by rhizosphere microbiota. Their findings suggest that *Lycopersicon esculentum* rhizosphere microbiota abundance decreased under exposure to diclofenac and ibuprofen, fungal/bacteria ratio decreased under exposure to diclofenac and ketoprofen, and carbon consumption rate (metabolic activity) increased due to diclofenac exposure. Furthermore, overhead irrigation lettuce shoots with water contaminated with pharmaceuticals (acetaminophen, caffeine, carbadox, carbamazepine, lincomycin, monensin sodium, oxytetracycline, sulfadiazine, sulfamethoxazole, trimethoprim and tylosin) resulted in higher abundance and diversity of ARGs on lettuce shoots [62]. Shen et al. [62] further highlighted that the overhead irrigation of lettuce shoots with water containing tylosin resulted in a microbial community with enriched Proteobacteria (specifically *Methylophilaceae*) and decreased alpha-bacteria diversity.

Despite all these reported consequences, it is important to have in mind that, in the environment, there is a complex and variable mixture of ECs (pharmaceuticals and non-pharmaceuticals) under different conditions (temperature, nutrients, pH, salinity, light, exposed community), which also constitutes a challenge to the understanding of real ecotoxicological risks of each ECs and to the identification of concerning ECs interactions. The effects of combinations/mixtures of chemical pollutants on environmental and human health are still underexplored since it is difficult to understand if the impact of one individual chemical will be potentiated or reduced in the presence of a complex and varying mixture of other compounds [63]. Also, information on ECs’ interactions in the environment is still scarce. An example of a concerning interaction is the adsorption of ECs on nanoparticles (NP) and microplastics (MP) since NP and MP can transport ECs (including pharmaceuticals), alter their toxicity and propensity to transformation, as well as their bioavailability, increasing the associated risks for the exposed communities [64,65].

### 3.1. Effects of Pharmaceutical Exposure on Aquatic Biofilms Behaviour

Surface-attached biofilms constitute the majority of microbial life in aquatic systems [66], and environmental biofilms are useful tools for assessing the impact of anthropogenic activities on aquatic ecosystems [67]. These environmental microbial communities have been widely used as bioindicators of the ecological status of aquatic systems whilst having a major role in biogeochemical cycles [66] and bioremediation processes for the removal of ECs [68]. Biofilms are complex microbial communities immobilized in a polymeric matrix and adhered to an inert or living solid surface [69]. Their polymeric matrix is composed of extracellular polymeric substances (EPS) secreted by the cells [70], consisting of polysaccharides and other biomolecules such as proteins, lipids, and nucleic acids [71]. EPS are the main ones responsible for ECs sorption processes on biofilms [72] as well as the interactions between pollutants and bacterial cell surface, for example, through the production of vesicles and other compounds that capture and/or solubilize ECs [5].

Induced changes in biofilm structure, metabolic function, composition and formation due to EC exposure have been brought up and observed by many authors [73,74,75,76,77,78,79,80]. Subirats et al. [79] concluded that the combination of nutrients and ECs (ciprofloxacin, erythromycin, sulfamethoxazole, diclofenac, and methylparaben) have a synergistic effect on bacterial composition, abundance and antibiotic resistance profile (increase of *sul1* and *intI1* ARGs) of artificial stream biofilms. On the other hand, long-term exposures to atenolol, carbamazepine, diclofenac, erythromycin, gemfibrozil, hydrochlorothiazide, ibuprofen, metoprolol and sulfamethoxazole have induced adverse effects on biofilms, specifically: a decrease on algal biomass and taxa richness, reduction on bacterial OTUs richness, decrease on diatoms and cyanobacteria abundance in comparison to green algae; increase on the metabolic processes rates, such as photosynthesis and community respiration, and bioaccumulation of pharmaceuticals (specifically: metoprolol, hydrochlorothiazide, sulfamethoxazole, carbamazepine and diclofenac) [71]. Moreover, Corcoll et al. demonstrated that all these effects were modulated by the flow intermittency [74]. Corcoll et al. [36] proved that the consequences from the presence of NSAIDs in fluvial biofilms (mixtures of ibuprofen and diclofenac from WWTP effluents) go beyond the acquisition of tolerance or resistance, suggesting that chronic pollution results in altered microbial metabolism since ibuprofen stimulated cyanobacteria abundance and growth rate and caused changes in the structure of algal communities, specifically, reduction of diatoms abundance (i.e., *Fragilaria* and *Cocconeis*) in favor of cyanobacteria abundance (i.e., *Oscillatoria* and *Chroococcales*)). Rosi-Marshal et al. [78] determined that pharmaceuticals (i.e., caffeine, cimetidine, ciprofloxacin, diphenhydramine and mixtures (caffeine + cimetidine + ciprofloxacin + diphenhydramine + metformin + ranitidine)) can alter biofilm bacterial community composition (increase of *Pseudomonas* sp. and decrease of *Flavobacterium* sp. relative abundance), suppress algal growth, microbial respiration in biofilms and photosynthesis. Proia et al. [81] found that ibuprofen and acetaminophen (paracetamol) negatively impacted photosynthesis and caused a decrease in the green algae/cyanobacteria ratio, while diclofenac was associated with higher phosphatase activity, whose effects on biofilm structure and function may have negative repercussions in river ecosystem functioning. Furthermore, Lawrence et al. [82] studied the effect of caffeine, carbamazepine, furosemide and ibuprofen on fluvial biofilms, concluding that these pharmaceuticals are responsible for the suppression of cyanobacteria. Similarly, Aubertheau et al. [67] also observed a reduction in cyanobacteria in river biofilms due to water contamination by WWTPs discharges (namely, WWTPs that use activated sludge followed by a reed-planted bed filter). The extinction of cyanobacteria (responsible for the production of O_2_ and CO_2_ fixation) could lead to catastrophic consequences for the maintenance of the global activity of the micro biosphere [83]. Since benthic river biofilms are the basis of the balance of aquatic ecosystems, any negative impact resulting from the presence of pharmaceuticals can affect biofilm-dependent trophic cycles and other relevant ecosystem processes [78].

The available literature regarding pharmaceuticals’ impact on fluvial biofilms overwhelmingly surpasses those relative to other aquatic environments. However, some studies provide insight into the effects of pharmaceutical ECs in microbial communities of different water bodies. Martin et al. [84] studied the effects of triclosan, at environmentally relevant concentrations, on marine periphyton biofilms, determining that its exposure results in changes in their bacterial composition. With increasing triclosan concentrations, *Rhodobiaceae* and *Rhodobacteraceae* families of *Alphaproteobacteria* and unidentified members of the candidate division *Parcubacteria* decreased their abundance, while families *Erythrobacteraceae* (*Alphaproteobacteria*), *Flavobacteriaceae* (*Bacteroidetes*), *Bdellovibrionaceae* (*Deltaproteobacteria*), several families of *Gammaproteobacteria*, and members of the candidate phylum *Gracilibacteria* increased in abundance. Shaw et al. [85] observed that exposure to pharmaceuticals impacted lentic biofilms (urban pond) respiration (due to exposure to diphenhydramine). Furthermore, diphenhydramine and ciprofloxacin suppressed algal biomass, and on the contrary, gross primary production was suppressed by diphenhydramine but stimulated by caffeine and cimetidine [85].

### 3.2. Effect of Pharmaceuticals on Drinking Water Associated Biofilms

DWDS are structurally, operationally, physicochemically and microbiologically diverse systems. Biofilm formation is inevitable in DWDS, forming an environment particularly rich in ecological niches where microorganisms can thrive and proliferate [86]. It is estimated that 95% of the total water biomass adheres to pipe surfaces (sessile state), while the remaining 5% is in a planktonic state [87]. Bacteria in biofilms of aquatic environments preferentially opt for a surface-associated state [88], as they display a higher tolerance to environmental adversities when compared to their vulnerable suspended counterparts [89]. Although the concentrations of ECs found in DW are very low, it is necessary to study the problems associated with prolonged exposures to determine potential changes induced by exposure to ECs in DWDS micro-ecosystems by quantifying their ecotoxicological repercussions and, consequently, the impact on the chemical and microbiological safety of distributed DW. As a consequence of significant changes, adverse effects may add concern to the existing problems related to the formation of biofilms in DWDS. Given the preference of bacterial communities to live in a sessile state, this topic has been heavily debated, as biofilms create a reservoir for pathogenic microorganisms that persist in DWDS and may be responsible for the incidence of water-borne diseases [90] and proliferation of other water quality-compromising bacteria (i.e., generating visual turbidity and alter organoleptic properties of DW), increase of hydraulic drag (compromising DW transport and distribution capacity), promotion of biocorrosion, decrease the efficiency of disinfection processes [91,92] and facilitate antibiotic resistance transfer [93]. Unquestionably, there is an interlinked relationship between water quality, DWDS microbiome and DWDS infrastructure integrity [92].

Research on ECs’ effect on DW microbial communities is still exceedingly limited. Only a few studies known to date were found to have sought to assess the effect of ECs on the DWDS surface-associated biofilms and whose objective was to determine the extent of EC exposure impacts [76,94,95,96,97,98]. However, only four of these studies included pharmaceuticals and represent just a small fraction of the total pharmaceutical ECs found in DW. Gomes et al. [95] determined that pre-exposure (for 26 days) of *Stenotrophomonas maltophilia* biofilms to mixtures of clofibric acid (170 ng/L) and carbamazepine (258 ng/L) increased the biofilm tolerance to removal by NaOCl. Consequently, Gomes et al. [96] studied the isolated effect of clofibric acid (for 12 weeks, at 170 ng/L) on *S. maltophilia* biofilms, resulting in the formation of biofilms with higher tolerance to chlorine disinfection and the antibiotic erythromycin. A better understanding of the extension of the biocidal action of disinfectants is extremely important to apply more adequate disinfection strategies and deliver efficient microbial control within DWDS [99]. The inefficiency of these treatments is of utmost concern since it hampers the biological control of DW and may constitute a biological hazard and contribute to the spread of antibiotic resistance and the selection and proliferation of ARBs [100,101]. Alternatively, Gomes et al. [94] reported that the inactivation of *Burkholderia cepacia* by sodium hypochlorite was hindered in the presence of caffeine (119 ng/L) and trimethoprim-sulfamethoxazole (1.7 and 8.2 ng/L, respectively). The effects of sulfadiazine and ciprofloxacin on DWDS biofilms were also studied by Wang et al. [76]. Their results suggest that the exposure of DWDS biofilms to sulfadiazine and ciprofloxacin increased 16S rRNA, ARGs and *int*1, as well as an increase in enzymatic activities and in the EPS production which, in its turn, promotes bacterial aggregation and adsorption on DWDS surfaces, and the dissemination of ARGs. Lastly, Zhang et al. [97] found that tetracycline promotes the growth of bacteria in biofilms, and exposure to tetracycline, sulfadiazine and chloramphenicol had enhanced chlorine and antibiotic resistance, and biofilm communities richness. These results show how unpredictable microbial behaviour can be faced with the presence of different pharmaceutical contaminants. Moreover, results described in the literature also emphasize the importance of understanding the impact of ECs on DWDS and environmental management.

Furthermore, assessment of the adverse biological effects of ECs, both individually and in the mixture (with additive, synergistic or antagonistic effects) [20,36,102], with chronic or acute exposures, may lead to the need to re-establish preventive actions, either by implementing restrictions on emissions (as most ECs are not regulated [20,36], or included in routine screening or monitoring programs [5,103]); by increasing the effectiveness of EC screening methods in DW; or by implementing more effective measures to eliminate ECs in aquatic systems [11,104]. The aggravation of contamination of various ecosystems is a scenario that is expected to increase in the coming years due to the increase in global population, urbanization, and the continued use of sources of contaminants, particularly in densely populated areas [5]. Additionally, climate change also poses a threat to water quality and quantity, as extreme weather conditions (e.g., rainstorms, flooding or droughts) will likely lead to DW production relying on unconventional and alternative water sources, as well as advanced treatment technologies [93].

### 3.3. The Role of Antibiotics and Non-Antibiotics on Acquired Antibiotic Resistance

The acquisition and dissemination of antibiotic resistance are highly promoted in aquatic environments, as they are repeatedly exposed to anthropogenic activities [105]. Antibiotic resistance can be intrinsic (of natural and predictable occurrence) or extrinsic/acquired (unpredictable evolution from genetic mutations/changes) [106]. Also, the resistance of bacteria in biofilms is attributed to a combination of the physical barrier composed of the EPS matrix and physiological resistance (due to genetic modification) [92]. These mutations can be acquired through the horizontal transfer of genes from different species [79,105,106,107]. Once resistance is acquired, cells divide and multiply, spreading exponentially, resulting in their rapid proliferation in aquatic systems [106,107]—vertical transfer of genes. Thus, the antibiotic resistance spread constitutes a concern regarding public health. The occurrence and development of antimicrobial resistance due to trace concentrations of ECs have long and recently been reported in either water (including marine and DW environments) or soil environments [62,76,104,108,109,110]. Increased antibiotic resistance may result in enhanced virulence, pathogenicity, disease outbreaks and transmission, as the consumer is directly exposed to antibiotic-resistant pathogens through the consumption of contaminated water [111].

The great popularity of antibiotics and their success in treating infections leads to these compounds being used excessively and inappropriately in non-clinical settings [112], accompanied by an increase in antibiotic release into the environment. Antibiotics exert not only toxic effects on microbial communities but also selective pressure [104,106,107,113], contributing to the continued selection of ARBs that contain ARGs (repressing susceptible species). This selective pressure represents a threat to public health [106] and constitutes an environmental issue. Additionally, ARGs and ARBs have also been considered as ECs. ARGs and ARG-bearing bacteria have been described in various aquatic environments, including DW, aquaculture surface water (fresh and marine waters), wastewater, and sediments [113,114,115]. Furthermore, biofilms are known to be hotspots for ARBs and ARGs proliferation [90,116], due to their high cell density, proximity, and accumulation of mobile genetic elements (MGE) [105]. Therefore, the need to assess the antibiotic impact on the development of resistance by biofilm communities is justified. Moreover, biofilm detachment contributes to the possible transport of ARB in DWDS [117].

It is commonly acknowledged that the overuse and release of antibiotics into the environment are key factors in the emergence of antibiotic resistance. It is known that sub-inhibitory concentrations are chemical cues that are major contributors to resistance promotion [118,119]. At concentrations below the minimum inhibitory concentration, antibiotics are unable to inhibit cell growth and promote cell death. Therefore it has been proposed that, in this situations, antibiotics act as signaling molecules that mediate various cell processes (such as gene transcription and expression, quorum sensing, inter- or intra-species communication, and biofilm formation, among others) [120]. For instance, Qiu et al. [113] also proposed that sulfamethoxazole exposure is positively correlated to the expression of ARG (*bla_d* gene) in microbial communities (*Fusobacteria*). Regarding the DW scenario, Wang et al. [76] proposed that the exposure to ciprofloxacin induced *mexA* (efflux pump encoding gene) expression and that the exposure to the combination of ciprofloxacin and sulfadiazine highly impacted the abundance of *mexA* and *intI1*. Furthermore, mixtures of pharmaceuticals have also been shown to have an impact on antibiotic resistance profiles, as mentioned by Subirats et al. [79] and previously in Section 3.1. However, little is known about the influence of non-antibiotics on the spread of antibiotic resistance in the environment [121]. Despite that, situations describing non-antibiotic ECs that exert selective pressure have been reported [121,122,123]. Non-antibiotic pharmaceuticals, such as ibuprofen, naproxen, diclofenac, the lipid-lowering drug gemfibrozil, and the β-blocker propranolol were described as able to accelerate the spread of antibiotic resistance through the uptake of exogenous ARGs [122].

Wang et al. [123] studied the effects of polycyclic aromatic hydrocarbons (naphthalene (100 mg/L) and phenanthrene (10 mg/L)) on the spread of antibiotic resistance in coastal microbial communities. Their results indicated that exposure resulted in a boost in the spread of ARGs, and this ECs presence induces a significant increase in *intI1*, *sulI* and *aadA2*. Additionally, certain metals (Cu (II), Ag (I), Cr (VI) and Zn (II), at subinhibitory concentrations) can select and stimulate antibiotic resistance (by promoting ARG transfer between *E. coli* strains), as revealed by Zhang et al. [124]. Lu et al. [125] evaluated whether exposure to environmental concentrations of the compound triclosan (0.2 to 20 µg/L) contributes to the spread of ARGs. Wang et al. [121] reported that CBZ (at concentrations of 0.05 mg/L) exposure increased ROS (reactive oxygen species) levels, SOS response, membrane permeability and the generation of *pilus*, which contributes to greater efficiency in resistance transfer. Furthermore, *Escherichia coli* K12 exposure to fluoxetine (5–100 mg/L) increases resistance against the antibiotics chloramphenicol, amoxicillin and tetracycline, and multiple resistance against fluoroquinolone, aminoglycoside, β-lactams, tetracycline and chloramphenicol [126]. Jin et al. [126] reported that antidepressant fluoxetine induces multiple antibiotic resistance in *E. coli* via the ROS-mediated mutagenesis (e.g., deletion, insertion, and substitution) of DNA-binding transcriptional regulators (e.g., *marR*, *rob*, *sdiA*, *cytR,* and *crp*) to further up-regulate the expression of efflux pumps (AcrAB-TolC pump jointly with the YadG/YadH transporter, the Tsx channel and the MdtEF-TolC pump). Similarly, Verma et al. [127] described that sodium salicylate (NSAID) and ibuprofen exposure increases *E. coli* antibiotic resistance by inducing the expression of transcription factors (*MarA*), consequently increasing the AcrAB-TolC efflux pump and *acrB* gene. The *Acinetobacter baylyi* exposure to ibuprofen, naproxen, diclofenac, the lipid-lowering drug, gemfibrozil, and the β-blocker propranolol is related to the enhancement of the transformation of ARGs (provided by promoted bacterial competence, enhanced stress levels, over-produced ROS and increased cell membrane permeability) [122]. These results prove that, even at environmentally relevant concentrations, multiclass ECs and pharmaceuticals, antibiotics or non-antibiotics, can promote the dissemination of ARGs and the acquisition of resistance by exposed microorganisms.

### 3.4. Controlling Pharmaceuticals Level in Water Sources and Wastewater

The consequences of the presence of pharmaceuticals in water sources cause global concern, and governmental entities have developed plans to improve knowledge in this field and to delineate actions to avoid ecological and public health consequences. For example, in 2019, the European Commission adopted the European Union Strategic Approach to Pharmaceuticals in the Environment, which focuses on different actions to address the environmental implications of human and veterinary pharmaceuticals, from design and production through use to disposal [128]. In this approach, several actions aimed to (1) increase awareness and promote prudent use of pharmaceuticals; (2) support the development of pharmaceuticals intrinsically less harmful to the environment and promote greener manufacturing; (3) improve environmental risk assessment and its review; (4) reduce wastage and improve the management of waste; and (5) expand environmental monitoring [128]. OECD also published a set of policy recommendations for the cost-effective management of pharmaceuticals ensuring the protection of water quality and freshwater ecosystems [129]. The proposed strategies are based on: (1) the improvement of knowledge, understanding and reporting on the occurrence, fate, toxicity, and human health and ecological risks of pharmaceutical residues in water bodies; (2) source-directed approaches (mainly targeted towards pharmaceuticals companies/manufacturing facilities) to encourage measures to prevent the release of pharmaceuticals into water bodies; (3) use-orientated approaches (close to physicians, veterinarians, pharmacists, patients and farmers) to encourage reductions in the inappropriate and excessive consumption of pharmaceuticals; (4) end-of-pipe measures to encourage improved waste and wastewater treatment to remove pharmaceutical residues after their use or release into the aquatic environment; and (5) implementation of life cycle approaches [129].

Both publications (from European Commission and OECD) are mainly based on the same fundamentals, improve knowledge on fate and risk assessment, improve stakeholders’ awareness, improve monitoring and improve water and waste treatment/management. A wide variety of technologies based on biological, chemical and physical processes are described in the literature as able to remove pharmaceuticals from wastewater and water sources. Among the biological processes, it is possible to enumerate the following as examples: activated sludge [130], microalgae treatment [131], biofiltration [132], and constructed wetlands [133]. Photoreaction can also be used to eliminate some pharmaceuticals, as reviewed by Abd Rahman, Choong [134], as well as filtration/adsorption processes [135,136] and advanced oxidation processes [137]. In this review, these strategies will not be discussed in detail since it is possible to find a vast number of recent review articles describing different strategies to remove pharmaceuticals and ECs in general [131,132,133,134,135,136,137].

## 4. Hardships in Assessing Potential Environmental Risks

There are still many knowledge gaps that cause numerous hardships and limitations in assessing the environmental risks of ECs, particularly the extrapolation of laboratory results with real systems [138] or between different aquatic environments. Particularly in the DW scenario, studies are scarce. Granted, aquatic ecosystems differ in their architecture, nutritive conditions, environmental stressors, and microbiological communities, as well as in substrates for biofilm formation [139]. Consequently, biofilm composition is variable depending on the environment in which they are inserted. Moreover, the response to different stress factors depends and varies from organism to organism, as well as interactions between flora and fauna (trophic interactions) [138]) and the presence of ECs (complexity of mixtures, exposure duration and concentration) that exert different actions on tested subjects. These limitations hamper the ability to predict and compare available results from studies performed in distinct environmental conditions.

Aside from the intricacy of the environmental conditions that influence the results obtained, there is a need to develop universal and standardized exposure systems where biofilm formation is supported for studying the possible outcomes of EC presence in aquatic environments, as well as methods for response characterization. Gomes et al. [5] highlighted the lack of standard tests to understand the extent of the impact of ECs on aquatic microbiomes and, consequently, the stability of the ecosystem, the biogeochemical cycle and the trophic chain. Some exposure models used in microbial ecotoxicology studies are artificial in nature when in situ studies are not applicable or recommended. This is also applied to the DW scenario, where the study of DW biofilms in real DWDS is a hard task since it may imply operational restrictions to DWDS, but also the high variability of operational conditions (water parameters, disinfection, hydrodynamics, temperature, pipe materials, among others) will hinder the reproducibility of the results between different experiments. Another problem associated with in situ DWDS biofilm formation studies is that the interior of the pipe network is not easily accessible due to the system’s closed infrastructure. Thus, acquiring biological samples through a non-invasive extraction method that readily provides or facilitates biofilm sampling representative of the spatial, temporal and physicochemical variation of real DWDS is challenging [91]. However, other devices have been described to carry out biofilm sampling in DWDS [140,141,142]. Overall, artificial enclosures or microcosms, carried out in controlled and monitored systems, that mimic local conditions of exposure to ECs (exposure time, nutrient load, flow dynamics, environmental or climatic conditions, and other stress factors, according to the region of impact) are preferred for primordial assessment of EC exposure effects, despite their limitations, to obtain reproducible results.

## 5. Conclusions

The presence of pharmaceutical contaminants in aquatic ecosystems and engineered water systems such as WWTPs and DWDS is unquestionable. However, most of the studies have been describing their presence in surface water and wastewater. These contaminants have a significant impact on microbial communities, specifically causing alterations in biofilm structure, metabolic function, composition and formation, which may be translated into alterations in the balance of nutrients in surface waters, soil and even in marine environments. However, these alterations may also result in microbiological problems in DW, specifically when bacteria proliferation is promoted by the presence of specific pharmaceutical contaminants or when ECs hinder DW disinfection.

The main worldwide concern about the impact of pharmaceuticals on environmental microbial communities refers to the impact of low concentrations of antibiotics that may impose selective pressure and contribute to antimicrobial resistance dissemination. However, evidence exists that the spread of antibiotic resistance elements is not exclusively caused by antibiotics. Non-antibiotic ECs, including some pharmaceuticals, such as some NSAIDs, at environmentally relevant concentrations have been related to the acquisition and dissemination of antimicrobial resistance.

Despite the recent advances reported in the literature on this topic, the experimental design still has some limitations that may compromise the comparison between studies and the achievement of conclusive findings. Therefore, this review highlights the importance of further studies on the impact of pharmaceutical residues in microbial communities and the development of adequate strategies to ensure that the exposure conditions mimic real scenarios.

## Data Availability

Not applicable.
